# Bortezomib Warhead-Switch Confers Dual Activity against Mycobacterial Caseinolytic Protease and Proteasome and Selectivity against Human Proteasome

**DOI:** 10.3389/fmicb.2017.00746

**Published:** 2017-04-27

**Authors:** Wilfried Moreira, Sridhar Santhanakrishnan, Brian W. Dymock, Thomas Dick

**Affiliations:** ^1^Department of Microbiology and Immunology, Yong Loo Lin School of Medicine, National University of SingaporeSingapore, Singapore; ^2^Department of Pharmacy, National University of SingaporeSingapore, Singapore; ^3^Public Health Research Institute, New Jersey Medical School, Rutgers, The State University of New JerseyNewark, NJ, USA

**Keywords:** TB, selective inhibitors, ClpP1P2, Bortezomib

## Abstract

Mycobacteria harbor two main degradative proteolytic machineries, the caseinolytic protease ClpP1P2 and a proteasome. We recently showed that Bortezomib inhibits ClpP1P2 and exhibits whole cell activity against *Mycobacterium tuberculosis*. Bortezomib, a dipeptide with a boronic acid warhead, is a human proteasome inhibitor approved for cancer therapy. The boronic acid warhead of the compound has been shown to drive potency against both the human proteasome and ClpP1P2 protease. Selectivity for the bacterial ClpP1P2 protease over the human proteasome is lacking but needs to be achieved to move this new anti-tuberculosis lead forward. In this study we explored whether an alternative warhead could influence Bortezomib's selectivity. We synthesized an analog containing a chloromethyl ketone instead of the boronic acid warhead and determined potencies against the bacterial and human enzymes. Surprisingly, the analog retained activity against mycobacterial ClpP1P2 and was active against the mycobacterial proteasome, but was devoid of activity against the human proteasome. Interrogation of a set of chloromethyl ketone peptides identified three additional compounds similarly inhibiting both ClpP1P2 and the proteasome in the bacteria while leaving the human proteasome untouched. Finally, we showed that these compounds display bactericidal activity against *M. tuberculosis* with cytotoxicity ranging from acceptable to undetectable. These results suggest that selectivity over the human proteasome is achievable. Selectivity, together with dual-targeting of mycobacterial ClpP1P2 and proteasome makes this new scaffold an attractive starting point for optimization.

## Introduction

*Mycobacterium tuberculosis* is the causative agent of tuberculosis (TB) which remains the biggest bacterial killer throughout history. In 2015, alone there were 9.6 million recorded cases of TB infection with 1.5 million deaths. Each year, half a million new cases of multidrug resistant infections further compound the situation (World Health Organization, 2016). There is an urgent medical need for new drugs with new mechanisms of action. We recently developed a novel target mechanism-based whole-cell screen and identified Bortezomib (BZ) as an inhibitor of the caseinolytic protease ClpP1P2 in mycobacteria with whole-cell activity against *M. tuberculosis* (Moreira et al., 2015).

Caseinolytic proteases are serine protease complexes found in a wide range of bacteria (Frees et al., 2007; Brötz-Oesterhelt and Sass, 2014) and are involved in removal of partially synthesized and misfolded proteins. In mycobacteria, the complex is composed of two catalytic subunits ClpP1P2 (Akopian et al., 2012; Raju et al., 2012) and regulatory subunits (ATPases). Catalytic subunits form a degradative chamber in which proteolysis occurs while regulatory subunits recognize substrates and provide the energy for unfolding proteins that are to be degraded. As part of the control of proteome homeostasis, caseinolytic proteases are involved in the removal of aborted translation products. The tmRNA trans-translation system, a bacterial rescue system that frees ribosomes stuck during protein synthesis, tags partially synthesized proteins with a caseinolytic protease specific (SsrA) degradation peptide (Keiler, 2008). SsrA-tagged proteins are recognized by the caseinolytic protease and degraded. We took advantage of this mechanism and used this ClpP1P2 protease-specific degradation tag to develop a fluorescence-based synthetic phenotype in order to detect and measure intracellular ClpP1P2 inhibition. Using this approach we identified Bortezomib as the first mycobacterial caseinolytic protease inhibitor with whole-cell bactericidal activity and a promising lead candidate against TB (Moreira et al., 2015).

Bortezomib is an N-protected dipeptidyl-boronate of a Pyrazine-Phenylalanine-Leucine-Boronic acid sequence (Pyr-FL-BA) and is the first proteasome inhibitor approved for the treatment of multiple myeloma and mantle cell lymphoma (Kane et al., 2006, 2007; Chen et al., 2011). Similar to ClpP1P2, the human proteasome is a protease complex composed of two main subunits, α and β, forming the proteolytic core (Da Fonseca et al., 2012). It has been shown via co-crystallization that Bortezomib forms a covalent adduct with the catalytic hydroxyl group of the eukaryotic proteasome active site residues (Schmitz et al., 2014). This leads to enzyme dysfunction, cell-cycle arrest and apoptosis in cancer cells (Bonvini et al., 2007). In contrast to most bacteria which do not harbor a proteasome, *M. tuberculosis* encodes a proteasome (Lin et al., 2006). Lin et al. have shown that Bortezomib inhibits the TB proteasome (Lin et al., 2009). Genetic deletion studies have shown that the mycobacterial proteasome is dispensable for growth but critical for virulence and adaptation to stress (Darwin et al., 2003; Gandotra et al., 2007, 2010; Totaro et al., 2016). We have shown that Bortezomib's antibacterial whole cell activity relies on ClpP1P2 inhibition (Moreira et al., 2015). Structure-based modeling revealed that the boronic acid forms a covalent bond via its boron atom with the serine hydroxyl of the ClpP1P2 catalytic triad (Moreira et al., 2015). Despite this novel antibacterial mechanism of action, Bortezomib's high potency against the human proteasome precludes its direct use for tuberculosis therapy.

To enable progression of a ClpP1P2 inhibitor toward a clinical candidate, selective inhibition of the bacterial ClpP1P2 protease over the human proteasome is required. It has been shown that Bortezomib's boronic acid warhead drives its potency against the human proteasome (Adams et al., 1998). We speculated that protease inhibitors carrying a different warhead may retain activity against ClpP1P2 while losing potency against the human proteasome. Chloromethyl ketones form a distinct class of covalent irreversible serine protease inhibitors. The chloride adjacent to the ketone moiety creates an electrophilic site that can react with activated nucleophiles, similarly to boronic acids (Bogyo and Wang, 2002). Despite their ease of synthesis, they displayed weak human proteasome inhibitory activities (Savory et al., 1993). We report here the observation that analogs of Bortezomib carrying a chloromethyl ketone warhead displayed selectivity in favor of mycobacterial ClpP1P2, while leaving the mammalian proteasome unaffected. Surprisingly, chloromethyl ketone derivatives showed activity against the mycobacterial proteasome.

## Materials and methods

### Compounds and chemistry

Details in Supplementary Material.

### Bacterial strains, mammalian cells, and culture conditions

*M. bovis* BCG (ATCC35734), *M. tuberculosis* H37Rv (ATCC 27294) and *M. bovis*-mRFP-SsrA strains were maintained in Middlebrook 7H9 media (Difco) supplemented with 0.5 % (v/v) glycerol, 0.05% (v/v) Tween 80, and 10% (v/v) Middlebrook ADC (Albumin-Dextrose-Catalase) (Difco). Hygromycin B (Roche) was added when appropriate. Enumeration of bacteria was performed by plating on Middlebrook 7h10 (Difco) agar plates containing 0.5% (v/v) glycerol, and 10% (v/v) Middlebrook OADC (Oleic acid-Albumin-Dextrose-Catalase) (Difco). HepG2 cells (ATCC HB-8065) were cultured at 37°C with 5% CO_2_ atmosphere in DMEM media (Gibco) complemented with 10% FBS heat-inactivated (Gibco), penicillin (100 U/mL, Gibco) and streptomycin (100 μg/mL, Gibco).

### *M. bovis-mRFP-SsrA* and ClpP1P2 inhibition assay

The plasmid pGMEH-p38-mRFP-SsrAec3 (Hygro^R^) carries the mCherry RFP gene cloned downstream of the p38 mycobacterial promoter and fused to the ClpP1P2-specific SsrA tag as well as a hygromycin-resistance cassette. pGMEH-P38-che-ssrAec3 was a gift from Dirk Schnappinger (Addgene plasmid # 27059). The plasmid was electroporated into *M. bovis* BCG to generate *M. bovis*-mRFP-SsrA. Transformants were recovered on 7H10 agar supplemented with 50 μg/mL hygromycin (Sigma-Aldrich) and grown in 7H9 broth supplemented with the same concentrations of hygromycin. Pre-culture of *M. bovis*-mRFP-SsrA were then harvested at mid-log phase, diluted to OD_600_ 0.2 in complete 7H9 media and dispensed into 96-well plates (200 μL/well) in presence of two-fold serially diluted compounds. *M. bovis*-p38-mRFP-SsrA untreated samples were used as negative control whereas *M. bovis*-p38-mRFP-SsrA treated with Bortezomib was used as positive control. Fluorescence signal acquisition was carried out after 24 h of incubation using a M200 Pro plate reader (Tecan). Red fluorescence was acquired under excitation/emission at λ = 587/630 nm. Relative fluorescence units were plotted as a function of drug concentration. The maximum fluorescence value obtained with Bortezomib was taken as the maximum (i.e., 100%) of ClpP1P2 inhibition. ClpP1P2 IC_50_ (i.e., the concentration required to inhibit 50% of ClpP1P2 activity) was determined in three independent replicates.

### Minimum inhibitory concentration (MIC) and minimum bactericidal concentration (MBC) determination

Turbidity-based growth inhibition was performed to assess anti-mycobacterial potency of the compounds. *M. bovis* BCG or *M. tuberculosis* H37Rv pre-cultures were harvested at mid-log phase and diluted to OD_600_ 0.05 in complete 7H9 media. Bacterial suspensions were then dispensed in 96-well plate (200 μL/well) in presence of two-fold serially diluted compounds and incubated for 5 days at 37°C under shaking (100 rpm). Cells were manually resuspended and OD was measured at 600 nm on M200Pro plate reader (Tecan). Percentage of growth was determined as compared to untreated control and plotted as a function of drug concentration. MIC_50_ (i.e., the concentration that inhibit 50% of growth) was determined in three independent replicates. MBC was determined by colony forming unit (CFU) enumeration on agar. Briefly, 10^6^ CFU from mid-log phase pre-culture of *M. tuberuculosis* H37Rv were treated for 5 days with concentrations ranging from one to ten times the MIC_90_ (Minimum inhibitory concentration required to inhibit 90% of bacterial growth) of each compound. Treated cultures were then plated on agar and CFU counted after 3–4 weeks of incubation at 37°C. CFU from an untreated inoculum were enumerated in similar manner as a control. MBC_99_ (i.e., the concentration that induce a 100-fold reduction in CFU count) was recorded. MBC_99_ was determined thrice independently.

### Bacterial and mammalian proteasome inhibition assay

Cell-based CT-like peptidase assay was performed using the Proteasome-Glo™ Cell-Based Assay Reagent (Promega) according to manufacturer's guidelines. Briefly, HepG2 cells (10^4^ cells/well) or *M. bovis* BCG log phase cultures were treated with the indicated compounds for 2 h followed by incubation with the luminescent substrate for 10 min. Luminescence was detected with a Tecan M200 Pro plate reader. Relative luminescence (RLU) was plotted as a function of drug concentrations and Proteasome IC_50_ (i.e., the concentration required to inhibit 50% of the proteasome activity) was determined. Bortezomib was used as a positive control.

## Results

### A warhead switch from boronic acid to chloromethyl ketone is sufficient to introduce selectivity

**Figure 1:**
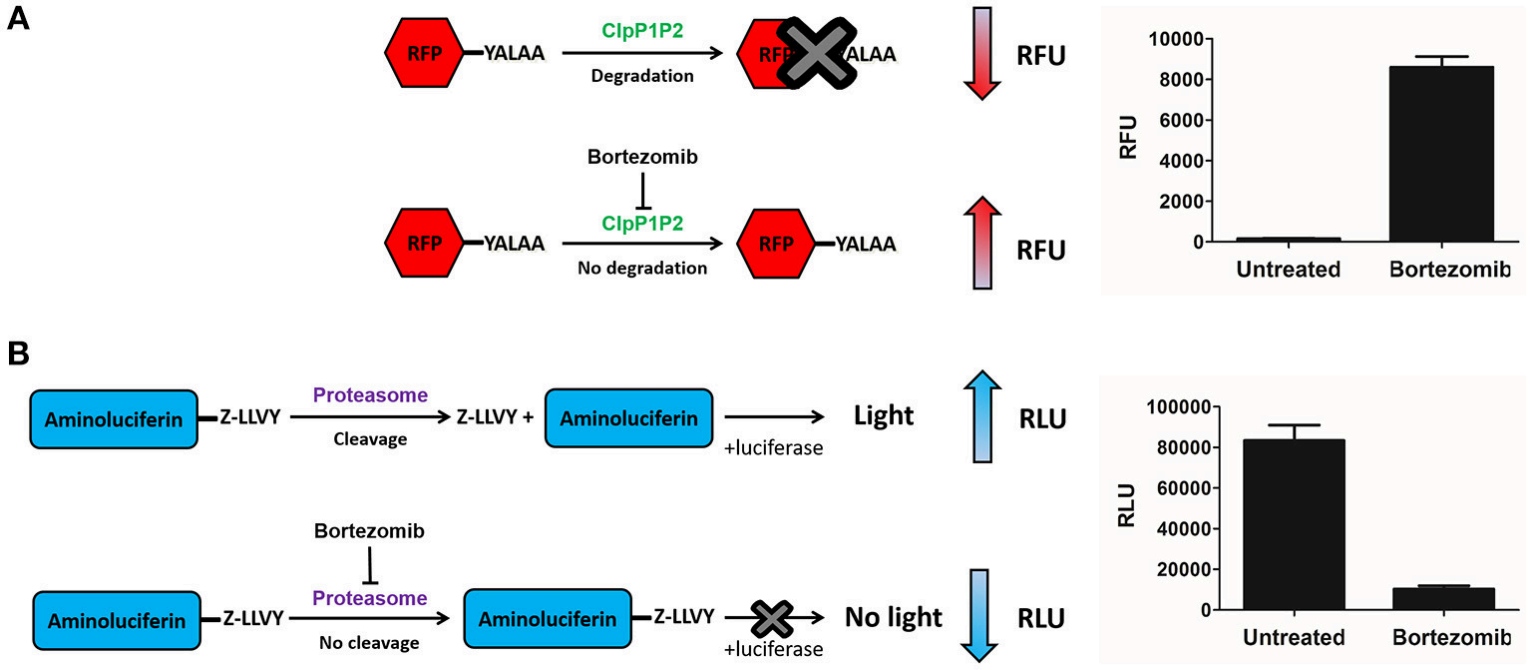
**ClpP1P2 and proteasome inhibition assays. (A)** ClpP1P2 inhibition assay principle. Under undisturbed conditions, ClpP1P2 recognizes and degrades SsrA-tagged (YALAA) RFP protein resulting in a low fluorescence level. In the presence of a ClpP1P2 inhibitor like Bortezomib, RFP is not degraded. Its accumulation results in an increase in fluorescence. **(B)** Proteasome inhibition assay principle. Under undisturbed conditions, the proteasome recognizes the Z-LLVY tag and cleaves it. The aminoluciferin is used as a substrate by the luciferase enzyme to generate luminescence. In the presence of a proteasome inhibitor like Bortezomib, the cleavage of Z-LLVY is prevented. The lack of luciferase substrate results in a reduced luminescence emission. RFU, relative fluorescence unit; RLU, relative luminescence unit.

Chloromethyl ketones (CMKs) comprise a distinct class of covalent serine protease inhibitors (Bogyo and Wang, 2002). We speculated that a Bortezomib analog carrying an alternative CMK warhead may retain ClpP1P2 activity while reducing activity against the human proteasome. Thus, we synthesized a Bortezomib derivative replacing the boronic acid with a CMK warhead (Pyrazine-phenylalanine-leucine-chloromethyl ketone or Pyr-FL-CMK) and determined its potency against bacterial and human enzymes. To measure the intracellular ClpP1P2 inhibition, we employed a *M. bovis* BCG strain expressing a ClpP1P2-specific SsrA-tagged Red Fluorescent Protein (RFP) reporter as described previously (Moreira et al., 2015). This reporter strain allows the measurement of the intracellular inhibition of ClpP1P2 protease via the accumulation of the non-degraded tagged RFP protein (Figure 1A). Under undisturbed conditions, the ClpP1P2 complex recognizes the SsrA-tagged RFP and degrades the protein, resulting in low basal fluorescence. In presence of a ClpP1P2 inhibitor, RFP-SsrA is no longer degraded and accumulates, leading to increase in fluorescence. We report here the ClpP1P2 IC_50_, i.e., the drug concentration required to inhibit 50% of activity. Bortezomib was used as a positive control. Bacterial and mammalian proteasome intracellular inhibition was measured using the whole-cell target-based Proteasome-Glo assay (Promega). This assay makes use of a proteasome chemotryptic activity-specific LLVY-tagged aminoluciferin (Gandotra et al., 2010). Under undisturbed conditions, the proteasome cleaves the LLVY tag, allowing the luciferase to oxidize the aminoluciferin generating luminescence. In the presence of a proteasome inhibitor, the LLVY cleavage does not occur. The tagged aminoluciferin cannot be used by the luciferase enzyme, preventing the emission of light (Figure 1B). We report here the proteasome IC_50_, i.e., the concentration required to inhibit 50% of the proteasome activity as compared to an untreated control. Bortezomib was again used as a control of proteasome inhibition.

As shown in Table 1, the CMK analog of Bortezomib, Pyr-FL-CMK, retained its activity against the bacterial target ClpP1P2 with about a 15-fold loss of potency (25 vs. 1.6 μM). Surprisingly, it also retained its activity against the bacterial proteasome albeit with a similar loss of potency compared to Bortezomib (25 vs. 0.8 μM). In contrast, Pyr-FL-CMK was inactive against the mammalian proteasome when tested up to a concentration of 500 μM, while Bortezomib (Pyr-FL-BA) displayed a proteasome IC_50_ of 5 nM. Mycobacterial ClpP1P2 and proteasome inhibition translated into whole cell activity against both the *M. bovis* BCG reporter strain as well as the actual pathogen *M. tuberculosis* with growth inhibitory concentrations (MIC_50_) of 20 and 25 μM, respectively. Furthermore, Pyr-FL-CMK displayed bactericidal activity within two-fold of Bortezomib's MBC_99_ (the minimum bactericidal concentration required to kill 99% of the bacterial population) of 100 vs. 50 μM. Finally, we assessed cytotoxicity of both Bortezomib and its CMK analog against mammalian cells using a standard MTS assay (Riss et al., 2004). We report here the CC_50_ (cytocidal concentration required to kill 50% of the cells) for each compound. The CMK analog was not toxic to HepG2 cells at up to 500 μM while Bortezomib displayed a CC_50_ of 250 μM. These results show that replacing Bortezomib's boronic acid with a chloromethyl ketone was sufficient to confer selective inhibition of the bacterial target over the mammalian proteasome. Surprisingly, the CMK analog not only retained ClpP1P2 activity but also mycobacterial proteasome activity (Table 1). Bacterial whole-cell growth inhibition, bactericidal potency were also retained while cytotoxicity was limited (Table 1).

**Table 1:** **Bortezomib and peptidyl-chloromethyl ketones target and whole cell activities**.

**Compounds**		**Target activity (μM)**			**Whole-cell activity (μM)**			
**Names**	**Structure**	**Bacterial Clp**	**Bacterial Prot**.	**Human Prot**.	***M. bovis***	***M. tuberculosis***	***M. tuberculosis***	**HepG2**
		**IC_50_**	**IC_50_**	**IC_50_**	**MIC_50_**	**MIC_50_**	**MBC_99_**	**CC_50_**
Pyr-FL-BA^1^	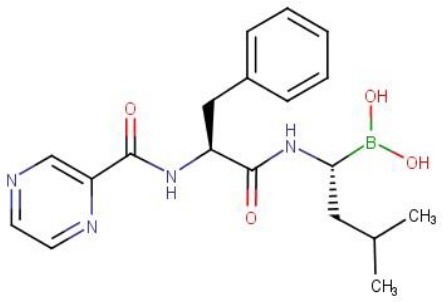	1.6	0.8	0.005	0.8	4	50	250
Pyr-FL-CMK	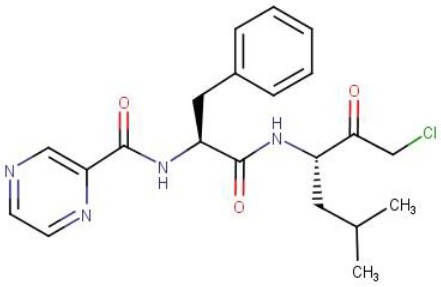	25	25	>500	20	25	100	>500
Z-GLF-CMK	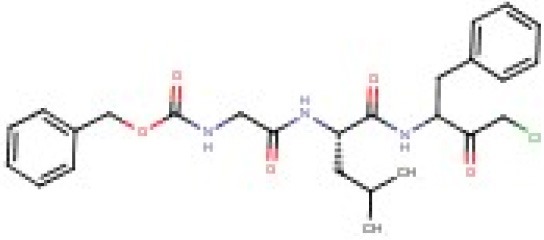	25	35	>500	20	20	200	60
Z-GGF-CMK	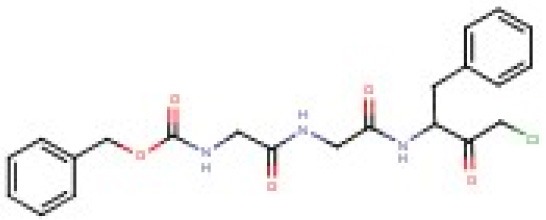	50	50	>500	30	30	nd	125

1 *Bortezomib; Pyr, pyrazine; BA, boronic acid; Z, carboxybenzyl; other compounds tested: Z-LY-CMK, Z-L-CMK, Z-F-CMK, Z-APF-CMK, Z-AAF-CMK, Z-FFR-CMK, Z-PFR-CMK, Z-LVG-CMK were found to be inactive in our assays. nd, not determined. Bacterial Clp IC_50_, concentration required to inhibit 50% of ClpP1P2 activity in M. bovis BCG. Bacterial Prot. IC_50_, concentration required to inhibit 50% of the proteasome activity in M. bovis BCG. Human Prot. IC_50_, concentration required to inhibit 50% of the proteasome activity in HepG2 cells. M. bovis, M. tuberculosis MIC_50_, concentration required to inhibit 50% of the growth of M. bovis or M. tuberculosis. M. tuberculosis MBC 99, concentration required to kill 99% of bacteria as compared to an untreated inoculum (100-fold kill). HepG2 CC50, concentration required to kill 50% of HepG2 cells as compared to an untreated inoculum. We report here the mean of three independent biological replicates. Standard deviations were <20%*.

To further support these findings, we interrogated a small library of 11 commercially available peptides harboring a chloromethyl ketone head group. We identified two CMK compounds active against mycobacterial ClpP1P2 and proteasome but inactive against the mammalian proteasome. As shown in Table 1, Carboxybenzyl-glycine-leucine-phenylalanine-CMK (Z-GLF-CMK) and Carboxybenzyl-glycine-glycine-phenylalanine-CMK (Z-GGF-CMK) inhibited ClpP1P2 and the proteasome at similar concentrations when compared to Pyr-FL-CMK while being inactive against the mammalian proteasome at concentrations up to 500 μM. For both compounds, target inhibition translated to whole cell bacterial growth inhibition. For Z-GLF-CMK, bactericidal activity was further characterized with an MBC_99_ of 200 μM (four- and two-fold higher than Bortezomib and Z-FL-CMK, respectively). However, it appeared that Z-GLF-CMK and Z-GGF-CMK both exhibited some cytotoxic activity against HepG2 cells with CC_50s_ of 60 and 125 μM, respectively. These compounds have been associated with serine protease inhibition, namely chymotrypsin, granzyme B, and cathepsin G (Bogyo and Wang, 2002) and they may have off-target effects which could account for their cytotoxicity. Nevertheless, these two compounds further support that peptidyl-chloromethyl ketone inhibitors display selective inhibition of both bacterial ClpP1P2 and proteasome targets.

## Discussion

We previously identified Bortezomib (Pyr-FL-BA), a potent human proteasome inhibitor, as an inhibitor of ClpP1P2 in mycobacteria with bactericidal activity against *M. tuberculosis*. In this study, we investigated whether selective inhibition of the bacterial ClpP1P2 protease over the human proteasome could be achieved. We tested the hypothesis that modifying Bortezomib's warhead may influence its selectivity. We showed that Pyr-FL-CMK, a chlorometyl ketone analog of Bortezomib, retained its activity against the bacterial ClpP1P2 protease while being devoid of activity against the mammalian proteasome. Mycobacterial ClpP1P2 belongs to the serine protease family. Chloromethyl ketones comprise a distinct class of covalent irreversible serine protease inhibitors. The function of this class of peptide electrophiles is mechanistically similar to that of boronic acids (Bogyo and Wang, 2002). The chloride adjacent to the ketone moiety creates an electrophilic site that can react with activated nucleophiles. Despite their ease of synthesis that prompted their development, these compounds display very weak, if any, inhibitory activity against the human proteasome (Savory et al., 1993). Interestingly, the CMK analog of Bortezomib showed activity against the mycobacterial proteasome while being inactive against the human proteasome, granting it an unprecedented selective dual targeting. The rather broad oligopeptide specificity of the mycobacterial proteasome has been previously documented (Lin et al., 2006, 2008) and might translate into an increased sensitivity to chloromethyl ketone inhibitors as compared to the human proteasome, against which this class of molecule performed poorly. Pyr-FL-CMK also remained bactericidal against *M. tuberculosis* with only a two-fold reduction in potency. Our findings that mycobacterial ClpP1P2 and proteasome selective inhibition translates into whole cell growth inhibition and bactericidal activity extended to two more CMK compounds (Z-GLF-CMK and Z-GGF-CMK), thus validating this strategy as a viable approach for drug discovery toward an anti-tuberculosis candidate. Finally, Pyr-FL-CMK was not cytotoxic to mammalian cells at concentration representing up to 20 times its MIC_50_ while for the two others CMK peptides selectivity was limited to three- to four-fold. This cytotoxicity could possibly be due to the inhibition of unidentified serine proteases. Altogether, this study represents a proof-of-concept establishing that selectivity for the mycobacterial ClpP1P2 protease over the human proteasome is achievable. CMK analogs of Bortezomib thus offer an attractive starting point for lead optimization that would aim at improving on-target potency while retaining their unexpected and unprecedented dual-targeting selective mechanism of action and low cytotoxicity.

## Author contributions

WM, BD, and TD designed the experiments and wrote the paper. WM carried out the biology, SS carried out the chemistry experiments.

### Conflict of interest statement

The authors declare that the research was conducted in the absence of any commercial or financial relationships that could be construed as a potential conflict of interest.

## Abbreviations

TB: tuberculosis
BCG: Bacille Calmette-Guérin.
