# Supplementary material for: Bortezomib Warhead-Switch Confers Dual Activity against Mycobacterial Caseinolytic Protease and Proteasome and Selectivity against Human Proteasome
Source: Front Microbiol. 2017 Apr 27;8:746. doi: 10.3389/fmicb.2017.00746 (PMC5406460; doi:10.3389/fmicb.2017.00746)
Supplement: Supplementary file 1 [file DataSheet1.DOCX]

**SUPPLEMENTARY MATERIALS**

Moreira et al., Bortezomib Warhead-Switch Confers Dual Activity Against Mycobacterial Caseinolytic Protease and Proteasome and Selectivity Against Human Proteasome

Source of compounds used and materials and methods used for chemistry

**Compounds**

Bortezomib was purchased from Selleckchem. All chloromethyl ketones compounds were purchased from Bachem.

**Chemistry**

Synthesis of pyrazine chloro ketone derivatives **8/9** are shown in scheme 1. Employing Ivanov’s procedure phenylalanine **1** was treated with *N,O*-bis(trimethylsilyl)-acetamide (BSA) which furnished **2**. Next, acylation of **2** with imidazolide **4**, synthesised from coupling of pyrazinoic acid **3** with imidazole, provided pyrazine acid **5** (Ivanov et al., 2009). Finally, coupling reactions were carried out between pyrazine acid **5** and chloro ketones [(*S*)-3-amino-1-chloro-5-methylhexan-2-one (**6**) (Taguchi et al., 1995) and (*S*)-3-amino-1-chloro-5-phenylpentan-2-one (**7**) (Weldon et al., 2014)] in the presence of 2-(1*H*-benzotriazole-1-yl)-1,1,3,3-tetramethyluronium tetrafluoroborate (TBTU) yielding *iso*propyl pyrazine **8** and phenyl pyrazine **9** respectively.

**Scheme 1. Synthesis chloro ketone derivatives 8/9***^a^*

|  | R |
| --- | --- |
| **6**, **8** |  |
| **7**, **9** |  |

*^a^* Reagents and conditions: (a) BSA, DCM, rt, 16 h; (b) CDI, DCM, rt, 16 h; (c) -40 °C to rt, 16 h; (d) **6**/**7**,TBTU, *i*Pr_2_EtN, DMF, 0 °C, 1 h.

**General Experimental Details**. All reactions requiring anhydrous conditions were carried out under a nitrogen atmosphere using oven-dried glassware (105 °C), which was cooled under vacuum. All reaction solvents used, such as dichloromethane, DMF were freshly collected from a solvent purification system. ^1^H (400 MHz) and ^13^C (101 MHz) with complete proton decoupling were performed on a 400 MHz Bruker Ultra Shield NMR spectrometer. Chemical shifts were reported as *δ* in units of parts per million (ppm) downfield from tetramethylsilane (*δ* 0.00), using the residual solvent signal as an internal standard: CDCl_3_ (7.26 ppm for ^1^H and 77.2 ppm for ^13^C). Multiplicities were given as s (singlet), d (doublet), t (triplet), q (quartet), m (multiplet), dd (doublet of doublets). Coupling constants (*J*) were recorded in hertz (Hz). All tested compounds were greater than 95% pure as determined by reverse phase high pressure liquid chromatography (HPLC) on a Shimadzu SPD-20A HPLC system. The test compound was dissolved in MeOH and injected through a 100 μL loop at a flow rate of 1.0 mL/min, with UV detection at 254 nm. Separation was carried out on a Zorbax SB-C18 column (250 mm × 4.6 mm, 5 μm, Agilent). Purity of each compound was assessed from the area of the major peak compared to total area of peaks obtained on the chromatogram. All commercial reagents were purchased from Sigma-Aldrich, Alfa Aesar, TCI and were of the highest purity grade available.

**Preparation of *N*-((*S*)-1-(((*S*)-1-chloro-5-methyl-2-oxohexan-3-yl)amino)-1-oxo-3-phenylpropan-2-yl)pyrazine-2-carboxamide (8)**

Under nitrogen, a solution of *i*Pr_2_NEt (0.23 mL, 1.30 mmol) in dry DMF (0.5 mL) was added drop wise to a suspension of ammonium salt **6** (57 mg, 0.29 mmol), pyrazine acid **5** (70 mg, 0.26 mmol) and TBTU (83 mg, 0.26 mmol) in dry DMF (3 mL) at 0 ^o^C. The stirring was continued for 1 h at 0 ^o^C. Then the reaction mixture was diluted with ethyl acetate. The organic layer was washed with 3% aqueous potassium carbonate, 3% aqueous citric acid, water and brine. The organic phase was dried over sodium sulphate and the solvent was removed under vacuum. The crude was purified by silica-gel (200-300 mesh) column chromatography using 50% ethyl acetate/hexane as eluting solvent to yield **8** as yellow solid (37% yield). ^1^H NMR (400 MHz, CDCl_3_) *δ* 9.29 (s, 1H), 8.69 (dd, *J* = 7.4, 2.4 Hz, 1H), 8.48 (s, 1H), 8.26 (d, *J* = 8.0 Hz, 1H), 7.32 – 7.12 (m, 5H), 6.19 (d, *J* = 7.3 Hz, 1H), 4.78 (dd, *J* = 14.7, 7.7 Hz, 1H), 4.68 – 4.59 (m, 1H), 4.03 (d, *J* = 1.9 Hz, 2H), 3.21 – 3.02 (m, 2H), 1.49 – 1.21 (m, 3H), 0.80 (d, *J* = 4.7 Hz, 3H), 0.78 (d, *J* = 4.8 Hz, 3H). ^13^C NMR (101 MHz, CDCl_3_) δ 200.7, 170.4, 163.1, 147.7, 144.4, 143.8, 142.8, 136.0, 129.3, 128.9, 127.4, 54.7, 54.6, 46.6, 40.0, 38.0, 24.8, 23.0, 21.5. LRMS-ESI (*m/z*): 417.17 [M + H]^+^.

Following a procedure similar that of **8**, *N*-((*S*)-1-(((*S*)-1-chloro-2-oxo-5-phenylpentan-3-yl)amino)-1-oxo-3-phenylpropan-2-yl)pyrazine-2-carboxamide (**9**) was prepared.

Yellow solid, yield: 26%. ^1^H NMR (400 MHz, CDCl_3_) δ 9.29 (d, *J* = 1.4 Hz, 1H), 8.70 (d, *J* = 2.4 Hz, 1H), 8.45 – 8.42 (m, 1H), 8.26 (d, *J* = 7.9 Hz, 1H), 7.27 – 7.06 (m, 9H), 7.00 – 6.97 (m, 1H), 6.44 (d, *J* = 7.3 Hz, 1H), 4.77 (dd, *J* = 14.9, 7.6 Hz, 1H), 4.64 – 4.59 (m, 1H), 3.94 (s, 2H), 3.12 (dd, *J* = 8.0, 5.8 Hz, 2H), 2.46 (t, *J* = 7.8 Hz, 2H), 2.17 – 2.02 (m, 1H), 1.84 – 1.72 (m,, 1H). ^13^C NMR (101 MHz, CDCl_3_) *δ* 200.1, 170.5, 163.2, 147.7, 144.4, 143.7, 142.8, 140.0, 136.0, 129.3, 128.9, 128.6, 128.3, 127.4, 126.4, 55.9, 54.7, 46.4, 38.0, 32.5, 31.4. LRMS-ESI (*m/z*): 465.27 [M + H]^+^.

**References**

Ivanov, A. S., Zhalnina, A. A., and Shishkov, S. V. (2009). A convergent approach to synthesis of bortezomib: the use of TBTU suppresses racemization in the fragment condensation. *Tetrahedron* 65, 7105–7108. doi:10.1016/j.tet.2009.06.042.

Taguchi, H., Yokoi, T., Tsukatani, M., and Okada, Y. (1995). Amino Acids and Peptides. XLI. 1:2 Facile Synthesis of 5-Methyl-2(1H)- pyrazinone Derivatives from Dipeptidyl Chloromethyl Ketones 3. *Tetrahedron* 51, 7361–7372. doi:10.1016/0040-4020(95)00385-L.

Weldon, D. J., Shah, F., Chittiboyina, A. G., Sheri, A., Chada, R. R., Gut, J., et al. (2014). Synthesis, biological evaluation, hydration site thermodynamics, and chemical reactivity analysis of α-keto substituted peptidomimetics for the inhibition of Plasmodium falciparum. doi:10.1016/j.bmcl.2014.01.062.
